# Utilization, Satisfaction, and Clinical Outcomes of People of Color and White Adults Using an Employer-Sponsored Digital Mental Health Platform

**DOI:** 10.3390/ijerph21121660

**Published:** 2024-12-12

**Authors:** Sara J. Sagui Henson, Camille E. Welcome Chamberlain, Brooke J. Smith, Jessica L. Jackson, Sharon L. Adusei, Cynthia M. Castro Sweet

**Affiliations:** Modern Health, San Francisco, CA 94108, USA

**Keywords:** digital mental health, health equity, race and ethnicity, depression, anxiety

## Abstract

Evaluating digital mental health services across racial and ethnic identities is crucial to ensuring health equity. We examined how People of Color (POC) and White adults were using and benefiting from an employer-sponsored digital mental health platform. A sample of 947 adults (42% POC) consented to an observational study and completed surveys on their identities and mental health outcomes at baseline and three-month follow-up. We examined care preferences, utilization, therapeutic alliance with mental health providers, and changes in outcomes among POC and White adults. At baseline, there were no race or ethnicity differences in preferred topics of focus (*p* = 0.36), rates of depression, anxiety, or loneliness (*p*s > 0.35), or self-reported well-being or stress (*p*s > 0.07). POC adults were more likely to prefer one-on-one care than White adults (*p* = 0.02). After 3 months of care utilization, there were no differences in therapeutic alliance (*p* = 0.52), use of therapy, coaching, or self-guided digital resources (*p*s > 0.47), or in the likelihood of improving, recovering, or maintaining clinical symptoms or psychosocial factors (*p*s > 0.07). Utilization, satisfaction, and clinical effectiveness were similar between POC and White adults, indicating the platform may offer comparable experiences. Evaluating utilization and outcomes among POC communities is necessary to inform consumers and help developers assess if innovations are fostering health equity.

## 1. Introduction

Despite the existence of health systems in place to provide mental healthcare in the United States, inequities across racial and ethnic groups persist in access, engagement, and clinical outcomes. It is well documented that People of Color (POC) experience worse mental health outcomes and are less likely to access mental healthcare than White people [1,2]. Among Americans with any mental health condition, White individuals have a higher rate of accessing care (49%) compared to Black/African American (31%), Native American/Alaska Native (41%), Native Hawaiian/Pacific Islander (28%), Hispanic/Latin(o)(a)(x) (33%), and Asian/Asian American (23%) individuals [3].

Disparities in accessing and engaging in mental healthcare partly stem from structural barriers rooted in racism that contribute to misdiagnoses, cultural insensitivity, and general mistrust of healthcare within communities of color [4]. Gaps in provider availability (especially POC clinicians), limited insurance coverage, and out-of-pocket costs also exacerbate the inaccessibility of mental healthcare for POC [3,5,6]. Unfortunately, disparities between the need for mental health services and connection to services have persisted in the past decade [7], worsened further by the disproportionate impact of the COVID-19 pandemic on communities of color [8]. To reduce disparities, we must focus on increasing mental health equity, defined as the state in which everyone has a fair and just opportunity to reach their highest level of mental health and emotional well-being [9].

One broad set of tools to address inequities in the provision of mental healthcare is through digital service delivery, which includes mobile health, health information technology, wearable devices, telehealth, and telemedicine [10]. The proliferation of digital health platforms has increased drastically over the last decade [11], spurred on by factors such as the COVID-19 pandemic, digital advancements, and increased financial investment in digital innovation [12,13]. Digital services are a crucial solution to providing accessible, equitable, and sustainable care, given their potential to reach wider populations in a more timely and less resource-intensive manner [14,15]. There is a body of literature supporting the effectiveness of digital mental health services for a range of mental health conditions, including depression, anxiety, substance use, and more [16,17]. Digital mental health platforms have the potential to solve critical gaps in care delivery if they integrate inclusive services without widening the digital divide or exacerbating existing health inequities.

However, when digital solutions do not involve POC groups to tailor services to different cultures, values, or language proficiencies, and when tele-mental health providers are not trained in cultural responsiveness, these solutions risk further excluding marginalized groups and reducing the acceptability and effectiveness of a digital solution. Prior research shows that culturally centered healthcare interventions reduce disparities and improve patient knowledge and health outcomes [18] and mental health interventions built with specific cultural adaptations are effective [15,19]. Digital programs must also consistently evaluate their mental health services for progress towards health equity, including investigating who is accessing and benefitting from services [9,20]. Research studies in digital health often lack the measurement of social identities and the representation of people from underserved communities that would enable evaluations of equitable engagement and effectiveness [21]. For example, one previous investigation found that most digital health studies did not include samples representative of the racial diversity in the United States [22]. Thus, some underserved populations may not benefit from innovation because they are excluded from intervention efficacy and effectiveness studies [23,24,25].

As an initial look at progress towards digital mental health equity, this investigation evaluated an employer-sponsored digital mental health benefits program that offers one-on-one sessions (therapy and coaching), self-guided digital resources, and group psychoeducation sessions through a web-based or mobile platform. Using Levesque et al.’s (2013) framework of healthcare accessibility [26], this program was built to be accessible, personalized, and culturally centered by addressing five dimensions of equitable service access and delivery: approachability (finding care), acceptability (culturally and socially appropriate care), availability (reaching care), affordability (paying for care), and appropriateness (engaging in effective care).

Services are provided as an employment benefit independent of other healthcare benefits, increasing the approachability of care and allowing for structural employer support in promoting and encouraging care connection. At the time this study was conducted, more than 1 million employees had access to mental healthcare through this employer benefit. Acceptability among employees with diverse needs and backgrounds was achieved by providing care recommendations that incorporate clinical symptoms, topics of focus, and care preferences. For one-on-one care, employees could access a provider network of therapists and coaches in 60+ countries and 55+ languages that were vetted to use evidence-based practices and culturally centered care. Digital content is translated into 13 languages and a Global Provider Inclusion Council advises the platform developers on country-specific support and culturally centered care.

Availability is ensured with evidence-based, on-demand digital resources, regularly available group support sessions, and/or one-on-one care matching using an algorithm that accounts for location, time preference, provider expertise, and provider identity (including filters for provider race and ethnicity). The platform continually monitors and adjusts provider availability to ensure the quickest time-to-first available session with a provider (currently <1 day). Affordability is enhanced with employer-sponsored access, meaning contracted services are free to employees. This reduces cost barriers and alleviates out-of-pocket spending on therapy/coaching that can range from hundreds to thousands of dollars per year. Appropriateness is evaluated by tracking engagement and effectiveness. In-house clinical researchers conduct studies linking engagement to clinical (e.g., clinical improvement and recovery, as well as maintenance of good mental health and prevention of worsening symptoms), organizational, and cost outcomes [27,28,29,30,31,32,33].

### The Present Study

Evaluating mental health programs for progress towards health equity is a necessary ethical principle [34] and crucial first step towards ensuring high quality, digital mental healthcare [35]. To evaluate this program, we examined differences in several outcomes among POC and White employees. First, because it is important to determine the characteristics of people using the program and what kinds of care they were seeking, we investigated race and ethnicity differences in baseline clinical presentation and care preferences. Second, we looked at race and ethnicity differences in utilization and therapeutic alliance, which are important indicators of mental health equity because when people are engaged with and satisfied with their care, they are more likely to experience beneficial treatment outcomes and complete the amount of care they need to improve [29].

Finally, we examined pre–post changes in clinical symptoms and psychosocial outcomes among POC and White adults. We chose to focus on more than symptom reduction because people may experience improvements to their mental health and quality of life beyond changes in depression and anxiety (e.g., enhanced well-being or diminished feelings of loneliness).

## 2. Materials and Methods

### 2.1. Design and Participants

This investigation was a prospective, longitudinal, observational study of adults newly registered for an employer-sponsored digital mental health benefits platform (Modern Health, San Francisco, CA, USA). Eligible participants were 18 years or older, living in the United States, had access to a smartphone, tablet, or computer, registered with the platform, and had matched with a therapist or coach and/or engaged with at least one element of digital content before enrolling in the study. This study protocol was reviewed and approved by Western Clinical Group Institutional Review Board. The study timeframe was 20 September 2021 through 31 May 2022.

### 2.2. Procedures

Information about the employer-sponsored digital mental health benefit was communicated to employees through various digital means, interpersonal interactions, and promotional activities within the workplace. The platform was provided at no cost to the employees in the study, as it was sponsored by their employer. Participation in the platform was voluntary. All participants in this study registered for an account on the mental health platform through a website or mobile app and completed onboarding assessments. Assessments collected participants’ care modality preferences (for example: one-on-one care, group care, self-guided digital content, etc.), topics of focus (for example: my emotions, my relationships, my physical well-being, etc.), and current clinical mental health status. The platform’s proprietary algorithm considered this information and recommended the participant to an initial care pathway. The recommendation was offered as an appropriate starting point based on the participants’ needs; they were not required to follow the recommendation and could self-refer to any form of care covered by their employer.

To recruit study participants, new registrants of the platform were sent an email invitation to participate in a study screening questionnaire approximately two weeks after onboarding. The invitation stated that this was a research study to learn more about the people who use the platform and the ways the services could benefit the participant and others. The screening questionnaire was designed to obtain demographic information to ensure a balanced sample across age groups, gender identities, race and ethnic identities, and clinical symptom severity at baseline.

After completion of the screening questionnaire, participants were invited to learn more about the study and provide informed consent to participate. Those who consented received a secure link to complete a baseline survey and a follow-up survey three months later. Surveys were hosted on a secure, online survey platform (Qualtrics, Provo, UT, USA). Participants were compensated with a USD 25 digital gift card upon completion of each survey. In addition to the information collected in the surveys, engagement metrics (such as, the number of therapy and/or coaching sessions attended, and the volume of digital content utilized) were collected directly from the mental health platform throughout the study period.

### 2.3. Digital Mental Health Services

The digital mental health platform was created with accessibility, inclusivity, and culturally centered care in mind, with the aim of achieving racial and ethnic equity across clinical outcomes. The platform’s comprehensive approach is informed by research on stratified stepped-care models [36,37] that provide all participants with the opportunity to receive high quality and evidence-based care tailored to their level of clinical need. Participants are given personalized care recommendations that integrate personal differences (such as clinical acuity, topics of focus, and care preferences). Recommendations may include working with a licensed therapist, an International Coaching Federation (ICF)-certified coach, self-guided digital resources, or group psychoeducation sessions. Participants could then access all these services on the home page of the platform.

#### 2.3.1. Therapy and Coaching

Therapy sessions were 50 min long and delivered by licensed clinicians (e.g., PhD—Doctor of Philosophy, PsyD—Doctor of Psychology, LCSW—Licensed Clinical Social Worker, LMFT—Licensed Marriage and Family Therapist, LPC—Licensed Professional Counselor). Coaching sessions were 30 min long and delivered by non-licensed providers with an International Coaching Federation (ICF) certification and at least 150 h of coaching experience. Sessions were delivered via videoconferencing on the web- or mobile-based platform. All providers were screened for their credentials and use of evidence-based practices, cultural humility, and ethics through written, interview, and practical assignments. Screening included reviewing responses to sample case studies, questions about the providers’ theoretical framework, and a sample visit evaluation.

Providers received additional training (at least 6 h) from Modern Health including didactics on culturally centered care, Modern Health’s proprietary model of care, and for coaches, evidence-based techniques (e.g., CBT—Cognitive Behavioural Therapy, ACT—Acceptance and Commitment Therapy, Motivational Interviewing, behavioral activation, and mindfulness) and how to assess high-risk situations that may require referring a participant to a therapist or crisis resource. Coaches also had ongoing case consultations with a licensed therapist. Culturally centered care training involved asynchronous programs or live webinars followed by case consultation hours, during which providers could meet with one another and the presenter to discuss and apply their learning. Example training topics included engaging in culturally centered practice, cultural humility, inclusive language, psychological safety, an introduction to stigma, and racial identity and racial trauma in clinical practice. Training was delivered by the clinical care team of psychologists, social workers, coaching specialists, and psychiatrists.

Ongoing provider quality monitoring and improvement was guided by a Quality Improvement Advisory Committee overseeing metrics such as member symptom improvement, therapeutic alliance ratings, post-session qualitative feedback, provider capacity and engagement, and time-to-first-available appointment. A Global Inclusion Council made up of mental health providers from around the world reviewed care delivery best practices for cultural nuance. Participants were given a list of providers with specialties matching their topics of focus (e.g., emotional, professional, social, physical, financial well-being) and could filter by provider race and ethnicity. The platform ensures providers in the network have the experience and training to support clients across demographics including race, ethnicity, gender identity, and sexual orientation. At the time of this study, the provider network of therapists and coaches spoke and delivered care in more than 55 languages. The number of sessions a participant completed varied based on clinical need and number of employer-covered free sessions. A total of 188 therapists and 363 coaches provided care to the participants in the current study.

#### 2.3.2. Self-Guided Digital Resources

Participants had unlimited, on-demand access to a library of evidence-based, self-guided digital educational and skill-building resources, including written content (i.e., interactive exercises and clinically backed skills), courses (i.e., self-paced, structured lessons developed and led by clinical therapists), podcast-style conversations about specific topics, meditations, breathing exercises, guided visualizations, and soundscapes. Resources covered many aspects of well-being including emotional (e.g., manage depression, reduce stress), professional (e.g., career growth, career changes, burnout), social (e.g., social identity in relationships, parenting skills, race and culture), physical (e.g., healthy lifestyles, sleep), and financial (e.g., finance distress, values) well-being. All resources were created by an in-house content studio production team including licensed clinical psychologists and subject matter experts with diverse racial, ethnic, geographical, and cultural backgrounds. The content studio adhered to inclusive language guidelines to ensure the content was relevant to as many participants as possible. At the time this study was conducted, resources were translated into 13 languages and representatives of the platform’s Global Inclusion Council reviewed content for cultural nuance (e.g., localized, non-stigmatizing mental health terminology and values).

#### 2.3.3. Group Psychoeducation Sessions

Participants had unlimited access to community-based spaces for learning and connection. Group psychoeducation sessions were live sessions led by licensed therapists and certified coaches from the platform network on a broad spectrum of topics ranging from foundational mental health subjects (e.g., emotional resilience) to global events (e.g., processing stressful world events), social justice issues (e.g., allyship), and community, identity-based, and affinity group sessions (e.g., POC men, Black individuals, Asian and Pacific Islander individuals). After initial creation, all sessions were audited by licensed clinicians to ensure that content was culturally sensitive and destigmatizing. Attendance was anonymous and session formats included webinar style learning, skill-building workshops, and informal discussions.

### 2.4. Measures

#### 2.4.1. Race and Ethnicity

Participants were able to select all that applied from the following list of racial and ethnic identities in the study screening questionnaire: American Indian or Alaska Native, Asian or Asian American, Black or African American, Hawaiian or Pacific Islander, Hispanic, Latino or Spanish origin, White, not Hispanic or Latino, and/or prefer to self-describe. For analyses, we grouped these identities as POC and White due to the smaller sub-group sample sizes for POC (see Section 3.1 Study Participants).

#### 2.4.2. Age

Participants provided their age in years in the screening questionnaire.

#### 2.4.3. Gender Identity

Participants were asked to select all that applied from the following list of gender identities: Agender, Genderqueer or genderfluid, Māhū, Man, Muxe, Non-binary, Questioning or unsure, Two-spirit, Woman, prefer to self-describe, and/or prefer not to say. For analyses, we grouped these identities as Woman, Man, and Non-binary.

#### 2.4.4. Education

Participants were asked to report their highest level of completed education from the following list on the screening questionnaire: high school or equivalent, vocational/technical school, Bachelor’s degree, Master’s degree, Doctoral degree, Professional degree, or prefer to self-describe. For analyses, we grouped these classifications as less than a Bachelor’s degree, Bachelor’s degree, and more than a Bachelor’s degree.

#### 2.4.5. Topics of Focus

Participants were presented with several topics of focus for seeking care in the platform. Topics were organized into five dimensions of well-being: “my emotions”, “my physical well-being”, “my relationships”, “my professional life”, or “my finances”.

#### 2.4.6. Care Preferences

At onboarding, participants were asked to select their preferred care modality from the following: “On my own”, “With a small group”, “One-on-one”, or “I’m not sure”. Care preferences were included in the care recommendation algorithm; for example, if a participant indicated they wanted to work one-on-one, they were recommended to begin care with a coach or therapist.

#### 2.4.7. Components of Therapeutic Alliance

After each session with a therapist or coach, participants had the option to respond to two adapted items from the Working Alliance Inventory [38] representing the agreement on goals and bond components of therapeutic alliance, respectively: “[My provider] and I are working on agreed upon goals” and “I am confident in [my provider’s] ability to help me”. Participants responded on a five-point scale (1 = Strongly Disagree; 5 = Strongly Agree). These response options were adapted from the Working Alliance Inventory’s original seven-point scale (1 = Never; 7 = Always). The number of survey items and response options was truncated to facilitate survey completion and encourage higher response rates from participants in a real-world setting. One score was computed for each participant with available data by averaging each participant’s response to these two items across all sessions within the three-month follow-up period. This assessment has shown appropriate reliability and validity previously [29], with a Cronbach alpha of 0.72.

#### 2.4.8. Engagement in Digital Mental Health Services

We tracked the number of therapy and coaching visits completed and the number of digital content items used during the three-month follow-up period. We did not have available data on the number of group psychoeducational sessions attended, as registration and attendance were anonymous.

#### 2.4.9. Depressive Symptoms

The nine-item Patient Health Questionnaire (PHQ-9) [39] was used to assess the presence and severity of depressive symptoms at baseline and follow-up. Participants responded on a four-point scale (0 = Not at all; 3 = Nearly every day). Item responses were summed to create a total continuous score ranging from 0 to 27, with higher scores indicating a greater severity of depressive symptomatology. A binary variable was created to indicate whether participants had a positive (PHQ-9 ≥ 10) or negative (PHQ-9 < 10) screen for depression at baseline [39]. Improvement in depressive symptoms was defined as a decrease of at least six points in the PHQ-9 score from baseline to three months [40]. Recovery was defined as a change from a positive screening at baseline to a negative screening at three months. Maintenance was defined as having a negative screening at both baseline and three months.

#### 2.4.10. Anxiety

The seven-item Generalized Anxiety Disorder Questionnaire (GAD-7) [41] was used to assess the presence and severity of anxiety symptoms at baseline and follow-up. Participants responded on a four-point scale (0 = Not at all; 3 = Nearly every day). Item responses were summed to create a total continuous score ranging from 0 to 21, with higher scores indicating a greater severity of anxiety symptomatology. A binary variable was created to indicate whether participants had a positive (GAD-7 ≥ 8) or negative (GAD-7 < 8) screen for anxiety at baseline [42]. Improvement in anxiety symptoms was defined as a decrease of at least four points in the GAD-7 score from baseline to three-month follow-up [43]. Recovery from anxiety symptoms was defined as a change from a positive screening at baseline to a negative screening at three months. Maintenance was defined as having a negative screening at both baseline and three-month follow-up.

#### 2.4.11. Loneliness

The three-item UCLA Loneliness Scale [44] was used to measure loneliness at baseline and three months. Participants responded on a three-point scale (1 = Hardly ever; 3 = Often) and item responses were summed to create a total continuous score ranging from 3 to 9. Higher scores indicated greater loneliness. A binary variable was also created to categorize participants as Lonely (total score > 5) or Not Lonely (total score ≤ 5). Recovery from loneliness was defined as a change in classification from Lonely at baseline to Not Lonely at follow-up. Maintenance was defined as no change in classification over time.

#### 2.4.12. Well-Being

The five-item World Health Organization Well-Being Index (WHO-5) [45] was used to measure well-being during onboarding and at three-month follow-up. Participants responded on a six-point scale (0 = At no time; 5 = All of the time). Item responses were summed and multiplied by four to obtain a total continuous score ranging from 0 to 100, with higher scores indicating better subjective well-being. A binary variable was created to indicate whether participants had lower (WHO-5 ≤ 50) or higher (WHO-5 > 50) subjective well-being, based on a clinically validated cut-off [45]. Improvement in well-being was defined as an increase of at least 10 points in the WHO-5 score from baseline to three-month follow-up [45]. Maintenance was defined as being above the threshold for higher well-being at baseline and three-month follow-up.

#### 2.4.13. Stress

The four-item Perceived Stress Scale (PSS-4) [46] was used to assess participants’ perceived level of psychological stress at baseline and three-month follow-up. Participants responded on a five-point scale (0 = Never; 4 = Very often). Total scores were calculated by summing all item responses to create a continuous variable ranging from 0 to 16, with higher scores indicating higher stress. A continuous score was used given that the PSS-4 does not have established clinical cut-offs or a threshold of clinically meaningful improvement.

### 2.5. Statistical Analyses

Data were analyzed using R Statistical Software (v4.2.2) [47]. Descriptive statistics were used to describe demographic characteristics and baseline care preferences. To test our first aim in the baseline sample, we used chi-squared tests to investigate the differences between POC and White participants in topics of focus, care preferences, and positive or negative screening for baseline depression, anxiety, and loneliness. We used independent-sample *t*-tests to look at differences in baseline well-being and stress scores.

To test our second aim in the follow-up sample, we used an independent-sample *t* test (Welch two sample *t* test due to unequal variances) to evaluate race/ethnic differences in therapeutic alliance. We also examined the percentage of participants with therapeutic alliance scores greater than or equal to four. We used independent-sample *t* tests to look at race/ethnic differences in engagement patterns among participants who engaged with at least one activity of a given care modality (i.e., differences in therapy use among those who had engaged in at least one therapy visit).

To evaluate our third aim exploring race/ethnicity differences in outcomes, we tracked changes in clinical symptoms and psychosocial outcomes relative to participants’ baseline symptoms. For participants with scores meeting the clinical cut-off for that measure at baseline (“higher risk”), we used logistic regression models with a binary race/ethnicity indicator (POC vs. White) as the predictor to evaluate the likelihood of improvement or recovery in that measure at follow-up. Specifically for depressive and anxiety symptoms, we looked at improvement or recovery; for loneliness, we looked at recovery; and for well-being, we looked at improvement. We also used chi-squared tests to compare differences in improvement/recovery rates. We present unadjusted models in the Section 3 Results and include models that adjust for age, gender identity, and level of education in the Supplementary Materials. We also present linear regressions using follow-up scores as the outcome, race/ethnicity as the predictor, and baseline scores as a covariate in the Supplementary Materials.

For participants that did not meet the clinical cut-off for that measure at baseline (“lower risk”), we used logistic regression models with race/ethnicity as the predictor and maintenance of none-to-mild symptoms of depression, anxiety, loneliness, and higher well-being as the outcomes. We used chi-squared tests to compare differences in rates of maintenance in each outcome. Because our stress measure did not have a clinical cut-off (to track recovery) or threshold of clinically meaningful improvement, we did not stratify stress analyses by baseline scores. Instead, we used a linear regression with race/ethnicity as the predictor and a change score representing follow-up stress scores minus baseline stress scores as the outcome.

## 3. Results

### 3.1. Study Participants

**Figure 1:**
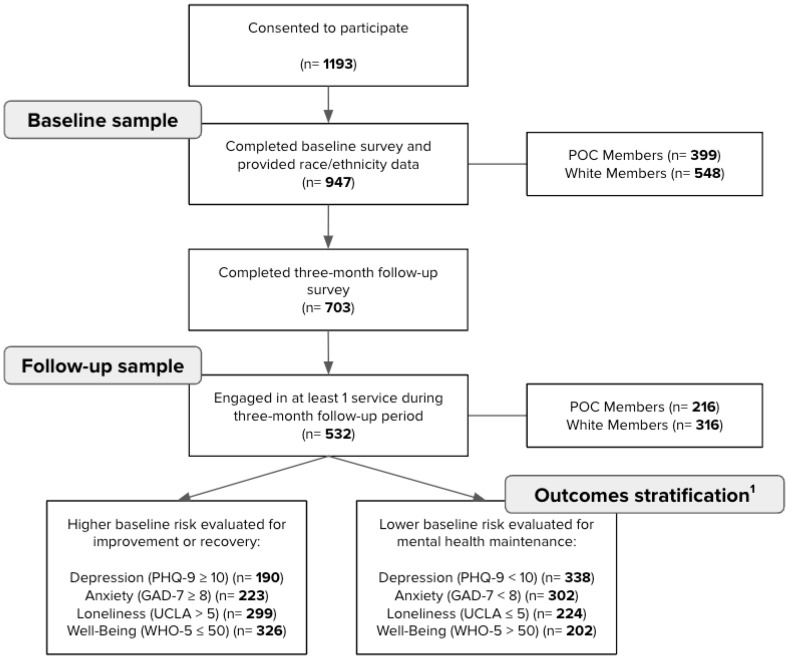
Flowchart of participants and stratification of outcome analyses. ^1^ Note: The stress measure did not have a clinical cut-off or threshold of clinically meaningful improvement; thus, we did not stratify stress outcome analyses by baseline scores.

Of the 8786 individuals who were eligible and invited to participate, 2032 completed the screening form, 1193 completed the consent form, and 950 enrolled and completed the baseline survey. Among this sample, three people were missing race/ethnicity data, leaving a baseline sample of 947 participants (see Figure 1). A total of 703 participants completed the three-month follow-up survey.

An analysis of baseline data on individuals who had complete follow-up data (“completers”, *n* = 703) compared to those without follow-up data (“non-completers”, *n* = 244) revealed no significant differences in race/ethnicity, age, gender identity, education, or percentage screening positive for depressive symptoms, anxiety symptoms, or loneliness (see Supplementary Table S1). Completers had higher levels of well-being (*p* = 0.003) and lower levels of stress (*p* = 0.004) than non-completers, a pattern consistent with broader research in which individuals with more complex needs are more likely to drop out of clinical research. However, both completers and non-completers had similar rates of depression, anxiety, and loneliness, indicating a diverse range of participants with substantial baseline risk that is representative of our target population.

Of the follow-up sample, 532 engaged in at least one of the digital mental health services during the three-month follow-up period and 171 were excluded because they did not engage in any service. This left a final sample of 532 participants available for outcomes analysis. An analysis of baseline data on individuals who had complete follow-up data and engaged (*n* = 532) compared to those with follow-up data who did not engage (*n* = 171) revealed no significant differences in race/ethnicity, age, gender identity, education, percentage screening positive for depressive symptoms, anxiety symptoms, or loneliness, or levels of well-being or stress. The participants were employed across 155 companies.

To address the study aims, baseline analyses were conducted using the sample of 947 participants and longitudinal analyses were conducted using the complete follow-up sample of 532 participants. At baseline, 548 (57.9%) participants identified as non-Hispanic White and 399 (42.1%) participants identified as POC, including 1 (0.1%) American Indian or Alaska Native, 184 (19.4%) Asian or Asian American, 57 (6.0%) Black or African American, 88 (9.3%) Hispanic, Latino, or Spanish origin, 2 (0.2%) preferred to self-describe, and 67 (7.1%) selected more than one race/ethnicity. Demographic characteristics of the baseline sample are reported in Table 1.

In the follow-up sample, 316 (59.4%) identified as non-Hispanic White and 216 (40.6%) participants identified as POC, including 1 (0.2%) American Indian or Alaska Native, 93 (17.5%) Asian or Asian American, 35 (6.6%) Black or African American, 47 (8.9%) Hispanic, Latino, or Spanish origin, 2 (0.4%) preferred to self-describe, and 38 (7.1%) selected more than one race/ethnicity. Additional demographic and baseline characteristics of the follow-up sample are reported in Supplementary Table S2.

### 3.2. Baseline Differences in Clinical Presentation and Care Preferences

See Table 2 for results on baseline differences between POC and White participants. There were no race/ethnicity differences in topics of focus (*p* = 0.36), average baseline well-being (*p* = 0.22, Cohen’s *d* = 0.08), or rates of screening positive for depression (*p* = 0.97), anxiety (*p* = 0.91), or loneliness (*p* = 0.35). POC adults were more likely to prefer one-on-one care than White adults (*p* = 0.02). Though not reaching statistical significance, POC adults reported slightly higher baseline stress than White adults (*p* = 0.07, Cohen’s *d* = 0.12).

### 3.3. Longitudinal Differences in Therapeutic Alliance and Engagement

Therapeutic alliance data were available for 223 participants who engaged in coaching and/or therapy sessions. There was no difference in average therapeutic alliance scores between POC (mean (*M*) = 4.58, standard deviation (*SD*) = 0.70) and White adults (*M* = 4.52, *SD* = 0.58), *t* (163.19) = 0.62, *p* = 0.53, Cohen’s *d* = 0.09. A total of 89.2% (*n* = 199) of participants reported therapeutic alliance ratings ≥ 4, with POC adults at 92.1% (*n* = 82) and White adults at 87.3% (*n* = 117).

Regarding engagement, 165 (31%) participants completed at least one therapy session, 302 (56.8%) completed at least one coaching session, and 362 (68%) used at least one digital resource. We defined duration of care as the number of days between a participant’s first and last service during the 3-month follow-up period. We found that for those engaged with therapy (who completed at least 1 session), the average duration of care was: *M* = 44.5 days, *SD* = 28.7 days. For those engaged with coaching (who completed at least 1 coaching session), the average duration of care was: *M* = 29.7 days, *SD* = 31.0 days. For those engaged with digital resources (who used at least 1 digital resource), the average duration of care was: *M* = 40.6 days, *SD* = 34.0 days.

Of the sample, 275 (51.7%) used one modality: 50 (9.4%) used therapy only, 98 (18.4%) used coaching only, and 127 (23.9%) used digital resources only. A total of 217 (40.8%) participants used two modalities: 22 (4.1%) used therapy and coaching, 53 (10%) used therapy and digital resources, and 142 (26.7%) used coaching and digital resources. Finally, 40 (7.5%) participants used therapy, coaching, and digital resources. There was no significant difference in the types of modalities used between POC and White participants (*χ*^2^ = 4.26, *p* = 0.64).

Among participants who engaged in at least one therapy visit (*n* = 165), there were no statistically significant differences in the average number of therapy visits for POC (*M* = 4.28, *SD* = 2.87, range: 1–12) versus White adults (*M* = 3.98, *SD* = 2.28, range: 1–9), *t* (133.05) = 0.72, *p* = 0.47, Cohen’s *d* = 0.12. Among participants who used at least one coaching visit (*n* = 302), there were no statistically significant differences in the number of coaching visits between POC (*M* = 2.47, *SD* = 2.03, range: 1–12) and White adults (*M* = 2.48, *SD* = 1.88, range: 1–12), *t* (300) = −0.04, *p* = 0.97, Cohen’s *d* = −0.01. Among participants who engaged with at least one digital resource (*n* = 362), there were no differences in the number of digital content engagements between POC (*M* = 3.69, *SD* = 4.35, range: 1–29) and White adults (*M* = 4.02, *SD* = 4.08, range: 1–22), *t* (360) = −0.72, *p* = 0.47, Cohen’s *d* = −0.08.

### 3.4. Longitudinal Changes in Clinical Symptoms and Psychosocial Outcomes

See Table 3 for all results regarding improvement, recovery, and change in clinical symptoms and psychosocial outcomes among participants who met the clinical cutoff for each measure at baseline. Logistic regressions (full results presented in Table 3) showed that there was no difference between POC or White adults in the likelihood of improving or recovering from depression (*p* = 0.60) or anxiety (*p* = 0.64). When comparing differences in improvement/recovery rates (chi-squared test results presented in-text), the percentage of POC adults who improved or recovered from depression (67.9%) was not significantly different from the percentage of White adults (64.3%) (*χ*^2^ = 0.14, *p* = 0.71), nor was the rate of improving or recovering from anxiety among POC (61.2%) or White adults (58%) (*χ*^2^ = 0.11, *p* = 0.74). There was no difference in the likelihood of recovery from loneliness (*p* = 0.21), with POC (21.1%) and White adults (27.6%) reporting similar rates of recovery (*χ*^2^ = 1.27, *p* = 0.26). There was no difference in the likelihood of improvements in well-being (*p* = 0.71), with POC (57.7%) and White adults (55.6%) reporting similar rates of improvement (*χ*^2^ = 0.07, *p* = 0.80). Finally, regardless of baseline symptoms, there was no race/ethnicity difference in the change in stress scores from baseline to follow-up (*p* = 0.33). The results of the regression analyses remained consistent after adjusting for age, gender identity, and level of education (see Supplementary Tables S3–S7).

See Table 4 for all results regarding maintenance in clinical symptoms and psychosocial outcomes among participants who had no/mild symptoms on each measure at baseline. Logistic regressions (full results presented in Table 4) showed that there were no race/ethnicity differences in the likelihood of maintaining none-to-mild depressive symptoms (*p* =0.77), none-to-mild anxiety symptoms (*p* =0.66), low levels of loneliness (*p* =0.07), or higher well-being (*p* = 0.39). Chi-squared test results (presented in-text) showed that the percentage of participants who maintained lower depression (*χ*^2^ = 0.01, *p* = 0.94), lower anxiety (*χ*^2^ = 0.07, *p* = 0.79), lower loneliness (*χ*^2^ = 2.70, *p* = 0.10), or higher well-being (*χ*^2^ = 0.42, *p* = 0.52) did not differ between POC and White adults. The results of the regression analyses remained consistent after adjusting for age, gender identity, and level of education (see Supplementary Tables S8–S11). We also ran a series of post-hoc linear regressions using follow-up scores as the outcome, race/ethnicity as the predictor, and baseline scores as a covariate and found there were no significant differences between POC and White participants in the overall point change on any outcome from baseline to follow-up (see Supplementary Tables S12–S16).

## 4. Discussion

Digital interventions that build upon a population health perspective hold great promise for addressing the current mental health crisis and the need for scalable solutions that can reach diverse communities. This study evaluated a technology-enabled mental health platform offered through employers that was built to be accessible and equitable to understand whether POC and White registrants had comparable rates of utilization, satisfaction, and clinical outcomes. Aligned with guidelines to integrate culture into both the human support and technological components levels [48], the platform employs a network of trained mental health providers from different cultural backgrounds who offer evidence-based practices and culturally centered care. Furthermore, culturally tailored self-guided resources developed by licensed clinicians and subject matter experts provided scalable access to people seeking technology-enabled non-one-on-one forms of mental health support.

Though there is much more work to be done, this study represents a first step in evaluating digital mental health equity among people from different racial and ethnic backgrounds. We purposefully examined satisfaction through ratings of therapeutic alliance components as an indicator of health equity because it contributes to patient activation [49] and can be hindered by healthcare provider biases and structural racism [50]. We also explored a variety of outcomes beyond clinical symptom reduction, as improvements in mental health may be experienced as more than reduced depression and anxiety for different groups. Investigating social aspects like loneliness, transdiagnostic skills like stress management, and the maintenance of good mental health among people starting care with no-to-mild symptoms are ways in which digital health solutions can evaluate whether patients experience mental health gains relevant to them and prevent escalating concerns.

Overall, we found far more similarities than differences in needs, preferences, care engagement, and clinical outcomes across POC and White participants. Our stratified recruitment sampling was successful in ensuring POC and White participants were similar across socioeconomic status and gender, though POC members were slightly younger by an average of two years. At baseline, there were no significant differences in individuals’ preferred topic of focus for care. We also saw no differences in baseline clinical symptom severity or the proportion who screened positive for possible depression, anxiety, or loneliness. The only statistically meaningful difference was a greater preference for working with a coach or licensed therapist one-on-one, with approximately 10% more POC participants preferring provider-based care relative to White participants. It is possible that POC members may have perceived a higher chance of being able to access more ethnically diverse providers through this platform than in previous healthcare interactions and so were more interested in one-on-one care options.

We also observed comparable utilization patterns across the different care modalities at follow-up. Overall, 31% of participants used therapy services, 57% used coaching services, and 68% used digital resources, which are high rates of uptake for digital health programs [51,52]. Across the sample, around 52% of participants used one modality of care and 48% used two or more modalities of care and average durations of care ranged from around 30–45 days depending on the modality.

Engagement in the content from the digital self-guided library was not significantly different; POC and White members similarly used an average of four pieces of digital content across the 12-week study period. Average session utilization was also not significantly different across therapy or coaching sessions, averaging around four therapy sessions and two to three coaching sessions within the three-month evaluation period. These comparable rates of utilization are encouraging given that previous evidence shows people who identify racially as White use significantly more tools within technology-enabled digital health interventions than people who are racially marginalized [53].

It is possible that these comparable rates of engagement are due to the platform’s use of Levesque et al.’s (2013) framework of healthcare accessibility [26]. Specifically, the acceptability and appropriateness dimensions of care were addressed by developing all digital content in-house with a team of licensed clinical psychologists and subject matter experts with diverse racial, ethnic, geographical, and cultural backgrounds. Additionally, the in-house content studio team adhered to inclusive language guidelines to ensure the content was as relevant for as many participants as possible. It is also likely that since content was translated into 13 different languages and reviewed by the platform’s Global Inclusion Council for cultural nuance (e.g., localized, non-stigmatizing mental health terminology and values), the offering was culturally centered and therefore participants were able to access content tailored for their needs.

Importantly, participants’ ratings of the therapeutic alliance with their coaches and therapists were quite high, with both groups rating their providers at an average score of 4.5 out of 5 across metrics of relationship bond and mutual therapeutic goals. Communities of color may experience greater challenges in establishing trust with mental health providers because of perceived or actual cultural differences or cultural biases [54,55]. It is imperative that people from all racial backgrounds be able to develop strong therapeutic relationships with their provider in a technology-enabled service given how that alliance impacts engagement, treatment retention, and outcomes [29,56]. Research has shown that finding a culturally appropriate fit such as racial and ethnic matching between clients and providers is associated with better therapeutic outcomes and therapeutic alliance [57]. Therefore, it is likely that having a network of therapists and coaches who were vetted for their use of evidence-based practices and culturally centered care, and with whom participants could match according to expertise and identity (including filters for provider race and ethnicity), was essential to achieving comparable therapeutic alliance outcomes across participant groups.

When examining clinical and psychosocial outcomes, we approached the data from a population health perspective and stratified the sample by baseline symptom severity. Those with symptoms that exceeded clinically validated thresholds were analyzed based on likelihood to improve or recover, while individuals with no to mild baseline symptoms were analyzed based on likelihood to maintain that low symptom level. Among those meeting clinically validated thresholds, we saw similar rates of improvement or recovery across measures of depression symptoms (64–68%), anxiety symptoms (58–61%), loneliness (21–28%), and well-being (56–58%) across POC and White participants. These rates are comparable to other stratified stepped care [58] and technology-enabled mental health interventions [59]. Likewise, we saw similar rates of mental well-being maintenance across all domains (with similarities ranging from 72 to 93%) across POC and White participants with no-to-mild baseline symptoms. The high rates of improvement and recovery, high rates of well-being maintenance, and the lack of significant differences based on racial and ethnic identity categories lend confidence that the platform is providing equitable clinical value across the spectrum of mental health needs.

### Limitations and Future Directions

As with most observational studies using “real-world evidence”, there are limits to the generalizability of our findings. The study was limited to adults who could access mental healthcare through their employer-sponsored benefits. Of the participants who expressed interest in the study by completing the screening form, 47% enrolled and completed the baseline survey. Though this may limit generalizability, this response rate is quite high for real-world observational studies which do not have as much control, and which recruit participants who are seeking mental healthcare through their employer and opting into a study for health outcomes monitoring (as opposed to enrolling in a research study to receive treatment). While we had a diversity of race/ethnic identities, the socioeconomic range was constricted, and thus we cannot generalize the findings to populations who access healthcare through self-funded or government-sponsored plans. Participation in this study was also voluntary; those who opted into the research may be different from those who accessed the platform but declined to participate in terms of their care preferences, mental health literacy, motivation to engage, or clinical presentation at baseline. We lacked detailed engagement data, including data on group psychoeducational sessions or frequency and temporal metrics of self-guided resource engagement.

We also did not recruit an equivalent volume of participants across the various race/ethnic subcategories. Our overarching recruitment goal was to reflect the current distribution of the platform’s population base. While the study’s recruitment of 42% of POC participants achieved that goal, the POC sample over-represents Asian/Asian American adults, and we did not have a sufficient volume of participants from any particular race or ethnic sub-category to conduct more discrete analyses. Future research should focus on achieving that aim and purposefully over-recruit enough participants in each race and ethnic group to avoid grouping participants into a POC group for comparison with White participants. Finally, our analytic approach aimed to test the probability of significant differences across race/ethnic groups; it does not confirm equivalence, only the lack of significant differences.

Future research can expand upon this work in several ways. First, to explore the regional variations in digital mental healthcare access and outcomes, subsequent work could investigate participants’ locations and potential geographic clustering effects. Next, collecting and analyzing qualitative data, particularly regarding satisfaction, could offer a richer understanding of participants’ experiences with digital mental healthcare. Although the focus of this study was on racial/ethnic differences in the effectiveness of shorter-term mental healthcare and outcomes (i.e., immediately post-treatment), future research should also explore longer-term engagement and maintenance of outcomes. Further, though we used clinically validated measures to ensure a robust assessment of each outcome of interest, future research could develop a tailored survey instrument with outcomes as distinct domains to streamline data collection. Finally, future research could offer the survey in multiple languages to increase accessibility and response rates.

## 5. Conclusions

Digital health companies are uniquely positioned to develop products and services that are equitable and do not perpetuate health disparities so long as they are providing culturally centered care and assessing mental health equity outcomes. Ensuring equity in digital health interventions is a necessary ethical principle. By having data on effectiveness for communities of color, patients, clients, and consumers from similar backgrounds can make informed decisions about whether a service is appropriate for them, reducing health data poverty. It is also essential to consider both individual and structural factors when evaluating accessibility and the effectiveness of digital mental health services. We encourage technology-enabled mental health solutions to continuously evaluate the outcomes of services as they are currently designed and delivered and keep adapting and tailoring them to meet differing needs. This includes considering factors such as health-related social needs, cultural preferences, values, health literacy, as well as adequate resources and representative datasets.

As more digital mental health solutions emerge in the healthcare landscape, it is imperative that these solutions are evaluated from an equity viewpoint to ensure they are providing value across various dimensions of diversity, and that they are not further compounding or exacerbating inequities. To ensure that digital health interventions are accessible and effective for all, our recommendation is that funders, regulators, and policymakers should require that interventions use culturally centered approaches. Such requirements would ensure that these interventions perform appropriately across different contexts and account for the diverse needs of patients, clients, and consumers, including communities that may not traditionally have access (i.e., those uninsured or publicly insured). It is critical to recognize digital interventions are not a one-size-fits-all solution; offering multiple care modality options, autonomy in dosing (e.g., weekly vs. monthly one-on-one sessions with digital supplement), and tailored services can help to meet the unique needs of different populations. By considering these factors, we can build digital interventions that not only address the current mental health crisis but also foster equity and reduce health disparities. As the world becomes increasingly digital, it is vital to ensure that digital health interventions prioritize equity and inclusivity.

## Figures and Tables

**Table 1:** Demographic characteristics of the baseline sample (*N* = 947) by race/ethnicity.

Demographic Characteristic	POC Members (*n* = 399)	White Members (*n* = 548)	*χ*^2^ or *t*	*p*
Age	32.38 ± 7.71	34.96 ± 9.22	−4.55	<0.001
Gender identity			5.77	0.06
Woman, *n* (%)	245 (61.6)	313 (57.2)		
Man, *n* (%)	129 (32.4)	213 (38.9)		
Non-binary, *n* (%)	24 (6.0)	21 (3.8)		
Education			0.94	0.62
<Bachelors, *n* (%)	64 (16.0)	76 (13.9)		
Bachelors, *n* (%)	224 (56.1)	320 (58.4)		
>Bachelors, *n* (%)	111 (27.8)	152 (27.7)		

Note: POC = People of Color.

**Table 2:** Baseline differences between POC and White participants in the baseline sample.

Variable	POC Members	White Members	*χ*^2^ or *t*	*p*
	(*n* = 399)	(*n* = 548)		
Topic of focus			4.37	0.36
Emotions, *n* (%)	193 (48.4%)	272 (49.7%)		
Professional life, *n* (%)	65 (16.3%)	75 (13.7%)		
Physical well-being, *n* (%)	35 (8.8%)	60 (11%)		
Relationships, *n* (%)	88 (22.1%)	125 (22.9%)		
Finances, *n* (%)	18 (4.5%)	15 (2.7%)		
Care preference			10.45	0.02
On my own, *n* (%)	31 (7.9%)	65 (12.2%)		
One-on-one, *n* (%)	264 (67.7%)	312 (58.4%)		
With a small group, *n* (%)	7 (1.8%)	19 (3.6%)		
I’m not sure, *n* (%)	88 (22.6%)	138 (25.8%)		
Positive depression screen, *n* (%)	145 (38.4%)	203 (38%)	0.001	0.97
Positive anxiety screen, *n* (%)	163 (43.1%)	234 (43.7%)	0.01	0.91
Positive loneliness screen, *n* (%)	213 (55.9%)	317 (59.3%)	0.89	0.35
Baseline well-being, *M* (*SD*)	44.16 (18.83)	42.67 (17.89)	1.24	0.22
Baseline stress, *M* (*SD*)	7.21 (2.95)	6.86 (2.85)	1.84	0.07

Note: *n* = 947. POC = People of Color. Positive/negative screens are based on clinical cut-offs for that measure. *M* = mean; *SD* = standard deviation.

**Table 3:** Likelihood of mental health improvement or recovery and percent improved or recovered between POC and White participants.

Outcome	*n*	B	SE	OR	95% CI	*p*	POC % Improved or Recovered	White % Improved or Recovered
Depression	190	−0.16	0.31	0.85	0.45–1.56	0.60	67.9	64.3
(PHQ-9 ≥ 10)								
Anxiety	223	−0.13	0.28	0.88	0.50–1.52	0.64	61.2	58
(GAD-7 ≥ 8)								
Loneliness	299	0.36	0.28	1.43	0.83–2.51	0.21	21.1	27.6
(UCLA > 5)								
Well-Being	326	−0.08	0.23	0.92	0.59–1.44	0.71	57.7	55.6
(WHO-5 ≤ 50)								
Stress	525	−0.23	0.23	n/a	n/a	0.33	n/a	n/a

Note: Sample sizes varied by analysis: POC *n*s = 78–130, White *n*s = 112–196. POC = People of Color; PHQ-9 = Patient Health Questionnaire-9; GAD-7 = Generalized Anxiety Disorder-7; UCLA = UCLA Loneliness Scale; WHO-5 = World Health Organization-5 Well-Being Index; B = standardized regression coefficient; SE = standard error; OR = odds ratio; CI = confidence interval. Race/ethnicity was the predictor in each model (POC coded as 0; White coded as 1).

**Table 4:** Likelihood of mental health maintenance and percent maintained between POC and White participants.

Outcome	*n*	B	SE	OR	95% CI	*p*	POC	White
							% Maintain	% Maintain
Depression	338	0.12	0.41	1.13	0.48–2.52	0.77	91.8	92.6
(PHQ-9 < 10)								
Anxiety	302	−0.16	0.35	0.86	0.42–1.69	0.66	88.1	86.4
(GAD-7 < 8)								
Loneliness	224	0.58	0.32	1.79	0.95–3.39	0.07	71.9	82
(UCLA ≤ 5)								
Well-Being	202	0.36	0.42	1.43	0.63–3.23	0.39	84.1	88.3
(WHO-5 > 50)								

Note: Sample sizes varied by analysis: POC *n*s = 82–134, White *n*s = 120–204. POC = People of Color; PHQ-9 = Patient Health Questionnaire-9; GAD-7 = Generalized Anxiety Disorder-7; UCLA = UCLA Loneliness Scale; WHO-5 = World Health Organization-5 Well-Being Index; B = standardized regression coefficient; SE = standard error; OR = odds ratio; CI = confidence interval; % maintain = percentage maintained. Race/ethnicity was the predictor in each model (POC coded as 0; White coded as 1).

## Data Availability

Individual de-identified data that underlie the results reported in this manuscript can be shared privately for research purposes upon receipt of a methodologically sound proposal and whose proposed use of the data from the study related to this article are approved by the authors. To gain access, requesters will need to submit a proposal to the corresponding author and sign a data access agreement that includes a commitment to the following: (1) using the data only for research purposes; (2) not attempt to, or actually, re-identify any individual; (3) securing the data using appropriate safeguards; and (4) destroying or returning the data after analyses are completed.

## Supplementary Materials

The following supporting information can be downloaded at: https://www.mdpi.com/article/10.3390/ijerph21121660/s1, Table S1: Baseline Differences between Completers and Non-Completers, Table S2: Demographic and Baseline Characteristics of the Follow-Up Sample (N = 532) by Race/Ethnicity, Table S3: Likelihood of Improvement or Recovery in Depression between POC and White Participants with Elevated Baseline Depression, Table S4: Likelihood of Improvement or Recovery in Anxiety between POC and White Participants with Elevated Baseline Anxiety, Table S5: Likelihood of Recovery in Loneliness between POC and White Participants with Elevated Baseline Loneliness, Table S6: Likelihood of Improvement in Well-Being between POC and White Participants with Lower Baseline Well-Being, Table S7: Changes in Stress between POC and White Participants, Table S8: Likelihood of Maintenance in Depression between POC and White Participants with Lower Baseline Depression, Table S9: Likelihood of Maintenance in Anxiety between POC and White Participants with Lower Baseline Anxiety, Table S10: Likelihood of Maintenance in Loneliness between POC and White Participants with Lower Baseline Loneliness, Table S11: Likelihood of Maintenance in Well-Being between POC and White Participants with Higher Baseline Well-Being, Table S12: Linear Regression Predicting Follow-Up Depression Scores between POC and White Participants, Table S13: Linear Regression Predicting Follow-Up Anxiety Scores between POC and White Participants, Table S14: Linear Regression Predicting Follow-Up Loneliness Scores between POC and White Participants, Table S15: Linear Regression Predicting Follow-Up Well-Being Scores between POC and White Participants, Table S16: Linear Regression Predicting Follow-Up Stress Scores between POC and White Participants.
