# Supplementary material for: Utilization, Satisfaction, and Clinical Outcomes of People of Color and White Adults Using an Employer-Sponsored Digital Mental Health Platform
Source: Int J Environ Res Public Health. 2024 Dec 12;21(12):1660. doi: 10.3390/ijerph21121660 (PMC11675968; doi:10.3390/ijerph21121660)
Supplement: Supplementary file 1 [file ijerph-21-01660-s001.zip › ijerph-3076634-supplementary.pdf]

Supplementary Materials

For manuscript "Utilization, Satisfaction, and Clinical Outcomes of People of Color and White Adults Using an Employer-Sponsored Digital Mental Health Platform"

Table S1. Baseline Differences between Completers and Non-Completers

**Baseline Characteristic**	**Non-Completers (n = 244)**	**Completers (n = 703)**	χ^2 **or** t	p
Race/ethnicity			0.73	.39
POC, *n* (%)	109 (44.7)	290 (41.3)		
White, *n* (%)	135 (55.3)	413 (58.7)		
Age, *M(SD)*	33.83 (8.43)	33.88 (8.81)	-0.08	.94
Gender identity			5.11	.08
Woman, *n* (%)	128 (52.9)	430 (61.2)		
Man, *n* (%)	101 (41.7)	241 (34.3)		
Non-binary, *n* (%)	13 (5.4)	32 (4.6)		
Education			4.02	.13
<Bachelors, *n* (%)	42 (17.2)	98 (13.9)		
Bachelors, *n* (%)	127 (52.0)	417 (59.3)		
>Bachelors, *n* (%)	75 (30.7)	188 (26.7)		
Positive depression screen	87 (41.6)	261 (37.1)	1.20	.27
Positive anxiety screen	103 (49.0)	294 (41.8)	3.15	.08
Positive loneliness screen	125 (58.7)	405 (57.6)	0.04	.84
Baseline well-being, *M(SD)*	40.26 (17.88)	44.35 (18.34)	-3.02	.003
Baseline stress, *M(SD)*	7.47 (3.09)	6.85 (2.81)	2.87	.004

Note. N = 947. POC = People of Color. Positive/negative screens are based on clinical cut-offs for that measure. Completers had significantly higher baseline well-being and lower stress.

Table S2. Demographic and Baseline Characteristics of the Follow-Up Sample (N=532) by Race/Ethnicity.

**Demographic or Baseline Characteristic**	**POC Members (*n* = 216)**	**White Members (*n* = 316)**	**χ^2 or *t***	***p***
Race/ethnicity				
American Indian or Alaska Native	1 (0.5)			
Asian or Asian American	93 (43.1)			
Black or African American	35 (16.2)			
Hispanic, Latino, or Spanish Origin	47 (21.8)		n/a	n/a
Self-describe	2 (0.9)			
More than one race/ethnicity	38 (17.6)			
non-Hispanic White	0 (0)	316 (100)		
Age, *M(SD)*	32.99 (8.06)	34.48 (9.10)	-1.95	.052
Gender identity				
Woman, *n* (%)	140 (64.8)	189 (59.8)	1.75	.42
Man, *n* (%)	66 (30.6)	114 (36.1)		
Non-binary, *n* (%)	10 (4.6)	13 (4.1)		
Education				
<Bachelors, *n* (%)	31 (14.4)	35 (11.1)	1.31	.52
Bachelors, *n* (%)	124 (57.4)	191 (60.4)		
>Bachelors, *n* (%)	61 (28.2)	90 (28.5)		
Topic of focus			1.39	.85
Emotions, *n* (%)	104 (48.1)	164 (51.9)		
Professional life, *n* (%)	35 (16.2)	41 (13.0)		
Physical well-being, *n* (%)	22 (10.2)	33 (10.4)		
Relationships, *n* (%)	48 (22.2)	67 (21.2)		
Finances, *n* (%)	7 (3.2)	11 (3.5)		
Care preference			3.02	.39
On my own, *n* (%)	14 (6.6)	34 (10.9)		
One-on-one, *n* (%)	143 (67.5)	195 (62.7)		
With a Small group, *n* (%)	4 (1.9)	6 (1.9)		
I'm Not sure, *n* (%)	51 (24.1)	76 (24.4)		
Positive depression screen	79 (36.6)	112 (35.4)	0.03	.86
Positive anxiety screen	86 (39.8)	138 (43.7)	0.63	.43

Positive loneliness screen	119 (55.1)	186 (58.9)	0.60	.44
Baseline well-being, *M(SD)*	45.11 (18.80)	43.80 (18.71)	0.79	.43
Baseline stress, *M(SD)*	6.98 (2.67)	6.72 (2.81)	1.04	.30

Note. POC = People of Color. Positive/negative screens are based on clinical cut-offs for that measure.

Table S3. Likelihood of Improvement or Recovery in Depression between POC and White Participants with Elevated Baseline Depression.

**Predictor**	***B***	***SE***	**OR**	**95% CI**	***p***
Intercept	0.07	0.90	1.07	0.18 - 6.28	.94
White	-0.29	0.33	0.75	0.39 - 1.42	.37
Age	0.02	0.02	1.02	0.98 - 1.06	.35
Woman	0.19	0.36	1.21	0.59 - 2.46	.59
Bachelor's Degree	-0.08	0.44	0.92	0.38 - 2.17	.86
> Bachelor's Degree	0.13	0.52	1.14	0.41 - 3.16	.80

Note. n = 180. POC = People of Color; B = standardized regression coefficient; SE = standard error; OR = odds ratio; CI = confidence interval. Race/Ethnicity was the predictor in each model (POC coded as 0; White coded as 1). Depression was measured with the PHQ-9. All participants in the analysis had baseline PHQ-9 $\geq$ 10. The reference groups for categorical covariates were: gender male, education < Bachelor's degree.

Table S4. Likelihood of Improvement or Recovery in Anxiety between POC and White Participants with Elevated Baseline Anxiety

**Predictor**	***B***	***SE***	**OR**	**95% CI**	***p***
Intercept	1.42	0.83	4.13	0.83 - 21.49	.09
White	-0.16	0.29	0.85	0.48 - 1.51	.58
Age	-0.01	0.02	0.99	0.95 - 1.02	.48
Woman	-0.32	0.32	0.73	0.38 - 1.37	.33
Bachelor's Degree	-0.32	0.42	0.72	0.31 - 1.62	.44
> Bachelor's Degree	-0.50	0.48	0.61	0.23 - 1.53	.30

Note. n = 212. POC = People of Color; B = standardized regression coefficient; SE = standard error; OR = odds ratio; CI = confidence interval. Race/Ethnicity was the predictor in each model (POC coded as 0; White coded as 1). Anxiety was measured with the GAD-7. All participants in the analysis had baseline GAD-7 $\geq$ 8. The reference groups for categorical covariates were: gender male, education < Bachelor's degree.

Table S5. Likelihood of Recovery in Loneliness between POC and White Participants with Elevated Baseline Loneliness

**Predictor**	***B***	***SE***	**OR**	**95% CI**	***p***
Intercept	0.32	0.83	1.38	0.27 - 7.11	.70
White	0.41	0.30	1.50	0.85 - 2.72	.17
Age	-0.05	0.02	0.96	0.92 - 0.99	.02
Woman	0.07	0.30	1.07	0.59 - 1.96	.83
Bachelor's Degree	-0.43	0.41	0.65	0.29 - 1.51	.30
> Bachelor's Degree	0.20	0.45	1.22	0.51 - 3.03	.65

Note. n = 279. POC = People of Color; B = standardized regression coefficient; SE = standard error; OR = odds ratio; CI = confidence interval. Race/Ethnicity was the predictor in each model (POC coded as 0; White coded as 1). Loneliness was measured with the UCLA Loneliness Scale. All participants in the analysis had baseline UCLA > 5. The reference groups for categorical covariates were: gender male, education < Bachelor's degree.

Table S6. Likelihood of Improvement in Well-Being between POC and White Participants with Lower Baseline Well-Being.

**Predictor**	***B***	***SE***	**OR**	**95% CI**	***p***
Intercept	-0.63	0.66	0.53	0.14 - 1.93	.34
White	-0.12	0.24	0.88	0.55 - 1.41	.61
Age	0.02	0.01	1.02	0.99 - 1.05	.26
Woman	0.24	0.26	1.27	0.77 - 2.11	.35
Bachelor's Degree	0.10	0.35	1.11	0.55 - 2.20	.77
> Bachelor's Degree	0.69	0.39	2.00	0.93 - 4.29	.07

Note. n = 312. POC = People of Color; B = standardized regression coefficient; SE = standard error; OR = odds ratio; CI = confidence interval. Race/Ethnicity was the predictor in each model (POC coded as 0; White coded as 1). Well-being was measured with the WHO-5. All participants in the analysis had baseline WHO-5 ≤ 50. The reference groups for categorical covariates were: gender male, education < Bachelor's degree.

Table S7. Changes in Stress between POC and White Participants.

**Predictor**	***B***	***SE***	**95% CI**	***p***
Intercept	-1.40	0.66	-2.69 - -0.11	.03
White	-0.22	0.23	-0.68 - 0.25	.36
Age	0.01	0.01	-0.12 - 0.04	.39
Woman	-0.39	0.25	-0.87 - 0.09	.11
Bachelor's Degree	0.82	0.37	0.10 - 1.53	.03
> Bachelor's Degree	0.31	0.39	-0.46 - 1.08	.43

Note. n = 502. POC = People of Color; B = standardized regression coefficient; SE = standard error; OR = odds ratio; CI = confidence interval. Race/Ethnicity was the predictor in each model (POC coded as 0; White coded as 1). Stress was measured with the PSS-4. Outcome was a change score representing follow-up stress scores minus baseline stress scores. The reference groups for categorical covariates were: gender male, education < Bachelor's degree.

Table S8. Likelihood of Maintenance in Depression between POC and White Participants with Lower Baseline Depression.

**Predictor**	***B***	***SE***	**OR**	**95% CI**	***p***
Intercept	1.61	1.27	5.03	0.41 - 63.44	.20
White	0.02	0.43	1.02	0.42 - 2.36	.97
Age	0.02	0.03	1.02	0.97 - 1.09	.37
Woman	-0.41	0.47	0.66	0.25 - 1.62	.39
Bachelor's Degree	0.13	0.68	1.14	0.25 - 3.90	.85
> Bachelor's Degree	0.92	0.80	2.51	0.47 - 12.17	.25

Note. n = 325. POC = People of Color; B = standardized regression coefficient; SE = standard error; OR = odds ratio; CI = confidence interval. Race/Ethnicity was the predictor in each model (POC coded as 0; White coded as 1). Depression was measured with the PHQ-9. All participants in the analysis had baseline PHQ-9 < 10. The reference groups for categorical covariates were: gender male, education < Bachelor's degree.

Table S9. Likelihood of Maintenance in Anxiety between POC and White Participants with Lower Baseline Anxiety.

**Predictor**	***B***	***SE***	**OR**	**95% CI**	***p***
Intercept	2.17	1.13	8.73	0.99 - 87.74	.06
White	-0.36	0.38	0.70	0.32 - 1.46	.35
Age	0.01	0.02	1.01	0.97 - 1.06	.54
Woman	-0.49	0.40	0.61	0.27 - 1.31	.22
Bachelor's Degree	-0.29	0.67	0.75	0.16 - 2.48	.67
> Bachelor's Degree	0.23	0.72	1.26	0.26 - 4.80	.75

Note. n = 290. POC = People of Color; B = standardized regression coefficient; SE = standard error; OR = odds ratio; CI = confidence interval. Race/Ethnicity was the predictor in each model (POC coded as 0; White coded as 1). Anxiety was measured with the GAD-7. All participants in the analysis had baseline GAD-7 < 8. The reference groups for categorical covariates were: gender male, education < Bachelor's degree.

Table S10. Likelihood of Maintenance in Loneliness between POC and White Participants with Lower Baseline Loneliness.

**Predictor**	***B***	***SE***	**OR**	**95% CI**	***p***
Intercept	0.61	0.99	1.84	0.26 - 13.14	.54
White	0.56	0.33	1.76	0.92 - 3.39	.09
Age	0.02	0.02	1.02	0.98 - 1.06	.40
Woman	-0.40	0.37	0.67	0.32 - 1.36	.28
Bachelor's Degree	-0.22	0.57	0.80	0.24 - 2.34	.70
> Bachelor's Degree	0.34	0.61	1.41	0.39 - 4.55	.58

Note. n = 221. POC = People of Color; B = standardized regression coefficient; SE = standard error; OR = odds ratio; CI = confidence interval. Race/Ethnicity was the predictor in each model (POC coded as 0; White coded as 1). Loneliness was measured with the UCLA. All participants in the analysis had baseline UCLA ≤ 5. The reference groups for categorical covariates were: gender male, education < Bachelor's degree.

Table S11. Likelihood of Maintenance in Well-Being between POC and White Participants with Higher Baseline Well-Being.

**Predictor**	***B***	***SE***	**OR**	**95% CI**	***p***
Intercept	1.68	1.45	5.37	0.32 - 105.47	.25
White	0.27	0.46	1.31	0.52 - 3.26	.56
Age	0.02	0.03	1.02	0.96 - 1.08	.56
Woman	-0.35	0.50	0.70	0.25 - 1.81	.48
Bachelor's Degree	-0.29	0.84	0.75	0.11 - 3.29	.73
> Bachelor's Degree	0.23	0.92	1.26	0.16 - 7.24	.80

Note. n = 193. POC = People of Color; B = standardized regression coefficient; SE = standard error; OR = odds ratio; CI = confidence interval. Race/Ethnicity was the predictor in each model (POC coded as 0; White coded as 1). Well-being was measured with the WHO-5. All participants in the analysis had a baseline WHO-5 > 50. The reference groups for categorical covariates were: gender male, education < Bachelor's degree.

Table S12. Linear Regression Predicting Follow-Up Depression Scores between POC and White Participants.

**Predictor**	***B***	***SE***	**95% CI**	***p***
Intercept	2.06	0.33	1.42 - 2.71	<.001
Baseline PHQ-9	0.48	0.03	-0.42 - 0.53	<.001
White	0.09	0.31	-0.52 - 0.69	.78

Note. n = 528. Outcome was the 3-month follow-up PHQ-9 score. B = standardized regression coefficient; SE = standard error; CI = confidence interval. Race/Ethnicity was the predictor in each model (POC coded as 0; White coded as 1). Depression was measured with the Patient Health Questionnaire-9 (PHQ-9).

Table S13. Linear Regression Predicting Follow-Up Anxiety Scores between POC and White Participants.

**Predictor**	***B***	***SE***	**95% CI**	***p***
Intercept	1.99	0.36	1.29 - 2.69	<.001
Baseline GAD-7	0.55	0.03	0.49 - 0.61	<.001
White	-0.07	0.34	-0.74 - 0.59	.83

Note. n = 525. Outcome was the 3-month follow-up GAD-7 score. B = standardized regression coefficient; SE = standard error; CI = confidence interval. Race/Ethnicity was the predictor in each model (POC coded as 0; White coded as 1). Anxiety was measured with the Generalized Anxiety Disorder Questionnaire-7 (GAD-7).

Table S14. Linear Regression Predicting Follow-Up Loneliness Scores between POC and White Participants.

**Predictor**	***B***	***SE***	**95% CI**	***p***
Intercept	1.64	0.20	1.24 - 2.04	<.001
Baseline UCLA	0.67	0.03	0.61 - 0.73	<.001
White	-0.14	0.12	-0.37 - 0.09	.24

Note. n = 523. Outcome was the 3-month follow-up UCLA score. B = standardized regression coefficient; SE = standard error; CI = confidence interval. Race/Ethnicity was the predictor in each model (POC coded as 0; White coded as 1). Loneliness was measured with the UCLA Loneliness Scale (UCLA).

Table S15. Linear Regression Predicting Follow-Up Well-Being Scores between POC and White Participants.

**Predictor**	***B***	***SE***	**95% CI**	***p***
Intercept	26.23	2.13	22.05 - 30.41	<.001
Baseline WHO-5	0.64	0.04	0.56 - 0.71	<.001
White	-0.60	1.51	-3.57 - 2.37	.69

Note. n = 528. Outcome was the 3-month follow-up WHO-5 score. B = standardized regression coefficient; SE = standard error; CI = confidence interval. Race/Ethnicity was the predictor in each model (POC coded as 0; White coded as 1). Well-Being was measured with the World Health Organization Well-Being Index (WHO-5).

Table S16. Linear Regression Predicting Follow-Up Stress Scores between POC and White Participants.

**Predictor**	***B***	***SE***	**95% CI**	***p***
Intercept	2.38	0.30	1.78 - 2.98	<.001
Baseline PSS-4	0.56	0.04	0.49 - 0.64	<.001
White	-0.33	0.21	-0.74 - 0.07	.11

Note. n = 525. Outcome was the 3-month follow-up PSS-4 score. B = standardized regression coefficient; SE = standard error; CI = confidence interval. Race/Ethnicity was the predictor in each model (POC coded as 0; White coded as 1). Stress was measured with the Perceived Stress Scale (PSS-4).
